# Charge Transport Mechanism in Thin Cuticles Holding Nandi Flame Seeds

**DOI:** 10.1155/2009/548406

**Published:** 2009-07-22

**Authors:** Wycliffe K. Kipnusu, Gabriel Katana, Charles M. Migwi, I. V. S. Rathore, Joshua R. Sangoro

**Affiliations:** ^1^Physics Department, Kenyatta University, P.O. Box 43844-00100, Nairobi, Kenya; ^2^Institute of Experimental Physics I, University of Leipzig, 04103 Leipzig, Germany

## Abstract

Metal-sample-metal sandwich configuration has been used to investigate DC conductivity in 4 *μ*m thick Nandi flame [Spathodea campanulata P. Beauv.] seed cuticles. *J*-*V* characteristics showed ohmic conduction at low fields and space charge limited current at high fields. Charge mobility in ohmic region was 4.06 × 10^−5^  (m^2^V^−1^s^−1^). Temperature-dependent conductivity measurements have been carried out in the temperature range 320 K < *T* > 450 K. Activation energy within a temperature of 320 K–440 K was about 0.86 eV. Variable range hopping (VRH) is the main current transport mechanism at the range of 330–440 K. The VRH mechanism was analyzed based on Mott theory and the Mott parameters: density of localized states near the Fermi-level N(*E*_*F*_) ≈ 9.04 × 10^19^  (eV^−1^cm^−3^) and hopping distance *R* ≈ 1.44 × 10^−7^ cm, while the hopping energy (*W*) was in the range of 0.72 eV–0.98 eV.

## 1. Introduction

A recent surge in interest in molecule-based electronics has highlighted the need for a better understanding of conduction mechanism in biomaterials [1]. Most biomaterials are obtained from plant products. Plant cuticle membranes have been found to contain high proportions of insoluble and acid resistant cutin biopolyester [2]. Current-voltage characteristics of Nandi flame seed cuticle biomaterial have shown electrical switching and memory properties [3]. Conducting biomaterials have been demonstrated to be potentially useful in making eco-friendly electronic devices and probes for sensors fabrication [4–7], solid electrolyte systems [8], barrier materials, and controlled release polymers [9], and in tissue engineering because they can easily be used as a platform that supports electrical stimulation of cell-tissue constructs [10]. 

 Generally charge conduction in semiconducting polymers is thought to take place by hopping of charge carriers in an energetically disordered landscape [11]. VRH conduction mechanism originally proposed by Mott for amorphous semiconductors [12] assuming a phonon-assisted process has been employed in previous studies [13–15] to explain charge transport in conducting synthetic polymers and their composites at low temperatures. Hopping charge transport has also been noted in double helix DNA [16] and in ion-doped biopolymers [8, 17]. 

While considerable time has been devoted to investigate conduction mechanism in synthetic polymers [18–23], only little attention has been paid towards electrical conductivity of pristine biopolymers. Consequently, there are notably few studies that can *reliably* explain conductance observations made with biopolymers [10]. Davis [1] suggested that electron and resonance tunneling is responsible for detectable charge transport in biomolecules sandwiched between metal electrodes. From measurements that probed changes in oxidized guanine damage yield with response to base perturbations, Armitage et al. [24] noted that charge transfer through base-base of DNA molecules takes place via the *π*-*π* bond overlap. Tao et al. [25] reported electron and bridge-assisted super-exchange charge transfer between donor and acceptor groups in peptide systems. Similar studies on proteins have also shown that electron transfer can occur across hydrogen bonds and that the rate of such transfer is greatly increased when the electron motions are strongly coupled with those of the protons [26]. Radha and Rossen [27] studied energy transport in biopolymers and suggested, based on the experimental results, that a soliton in biopolymers is an energy packet (similar to the “conformon” which is the packet of conformational strain on mitochondria) associated with a conformational strain localized in region much shorter than the length of a macromolecule. It was noted that as the soliton (localized curvature) moves on the polymer, it could trap an electron and drag it along. This mechanism may be important in understanding charge transport in biological molecules, where curvatures abound. 

In this paper, we report dc conductivity measurements and charge transport mechanisms in Nandi flame seed cuticle (a pristine biopolymer) in the temperature range 350–450 K. Fourier transform infrared (FT-IR) spectra analysis of pristine Nandi flame seed cuticle in comparison with FT-IR spectra of cellulose and surface probe analysis using atomic force spectroscopy (AFM) are presented before electrical conductivity. Discussion of charge transport mechanism is presented based on the fact that at least one mechanism can predominate within a specific temperature range. The following charge transfer mechanisms: electric field dependent Schottky and Poole-Frenkel emission, space charge limited current (SCLC) (from which some electrical parameters of the sample are obtained), tunneling current, and VRH are discussed in that order.

## 2. Experimental

**Figure 1:**
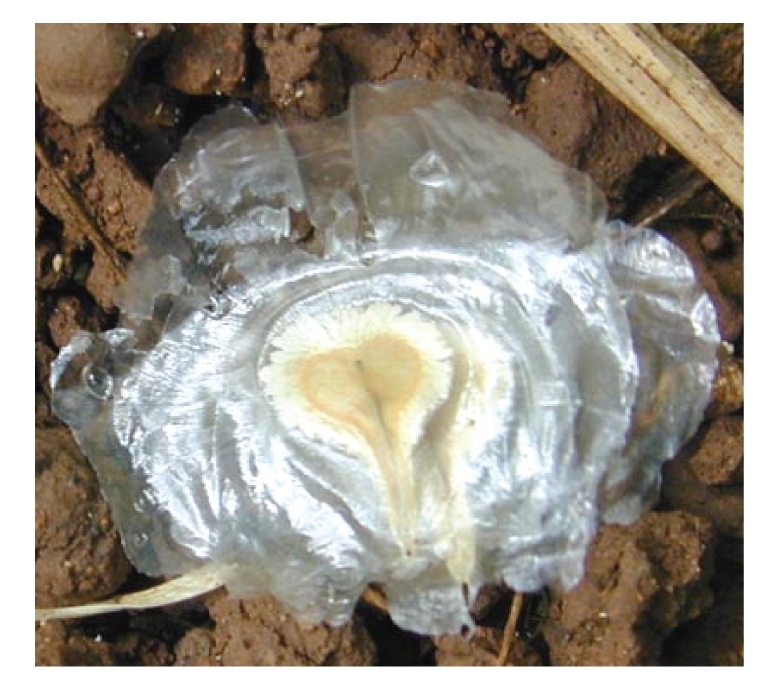
Translucent and thin cuticle holding Nandi flame seed.

 Samples of thin seed cuticles (Figure 1) were obtained from Nandi flame trees, also known as African tulip, which are used as ornamental trees in Kenya. Samples were well cleaned with acetone and dried at a temperature of 310 K for 12 hours inside a Lindberg blue tube furnace of model TF55035C and then stored in polythene bags containing silica gel desiccators. FT-IR studies were done in order to compare the IR spectrum of the cuticle to that of cellulose. Samples for FT-IR measurement were prepared by grinding the sample (0.1–2.0 percent by weight) with Potassium Bromide (KBr) and compressing the whole into a wafer. Thickness of the samples was determined by an interferometric method [28] and found to be about 4 *μ*m. Electrode coating on the film of pristine cuticles was done using quick drying and highly conducting flash-dry silver paint obtained from SPI Supplies, Pa, USA. A mask (made of stiff and thin transparent polythene paper) of circular aperture of 0.56 cm diameter was used while coating, to ensure uniformity in size of coated surface. Circular aluminum foil of the same diameter was placed on freshly coated surface such that the sample was sandwiched between two aluminum electrodes. These metal-cuticle-metal sandwiches were left to dry at room temperature for a period of 24 hours to ensure that there was good ohmic contacts between aluminum electrode and the sample cuticle. Flash-dry Silver paint was used to connect thermally insulated thin wires onto the aluminum electrodes. A cuticle sandwiched between aluminum electrodes was placed inside the Lindberg/Blue Tube Furnace and temperature varied at steps of 5 K between 350 K and 500 K at constant electric fields while measuring current-Voltage (*I-V*) using Keithley 2400 series source meter. 

Surface structural characterization of the pristine cuticles was done using AFM (a Digital Instrument Multimode/Nanoscope III scanning probe microscope was used). Taking images at the fundamental resonance frequency of Si cantilevers (≈300 kHz) while operating the instrument in the tapping mode, the height and phase images were obtained simultaneously.

## 3. Results and Discussion

**Figure 2:**
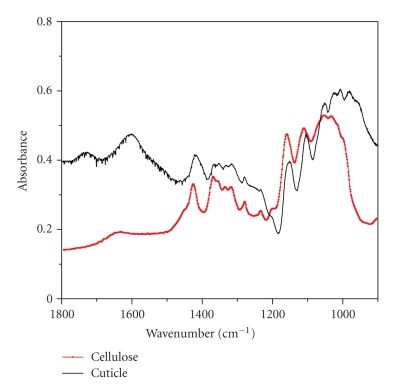
Absorbance FTIR spectra of the cuticle in comparison to that of cellulose.

The FT-IR spectra of the cuticle and cellulose are shown in Figure 2. The IR bands (given with accuracy of 2 cm^−1^) and band assignments are listed in Table 1. Cellulose IR data bands and comments in Table 1 are based on literature [29]. Vibrations in the 1800 to 1200 cm^−1^ range mostly arise from side chains or side groups (such as ester and OH) while vibration in the 1200 to 900 cm^−1^ are related to backbones of polysaccharides such as cellulose, hemicelluloses, and pectin [29]. The strong IR bands of the cuticle at 1278 cm^−1^ assigned to *δ* (CH), and 1106 cm^−1^ assigned to *ν*(CO), *ν*(CC) are overlapped with strong cellulose bands at 1277 cm^−1^ and 1107 cm^−1^, respectively. Generally, the cuticle IR bands in the range 1050 to 1400 cm^−1^ are strongly correlated with the bands of cellulose. This therefore means that the cuticle backbone molecules are composed of cellulose. However, IR absorption peaks of the cuticle in the carbonyl side group region (1750–1600 cm^−1^) and in the region related to backbone of polysaccharides (1025–950 cm^−1^) do not correspond to cellulose bands. 

**Figure 3:**
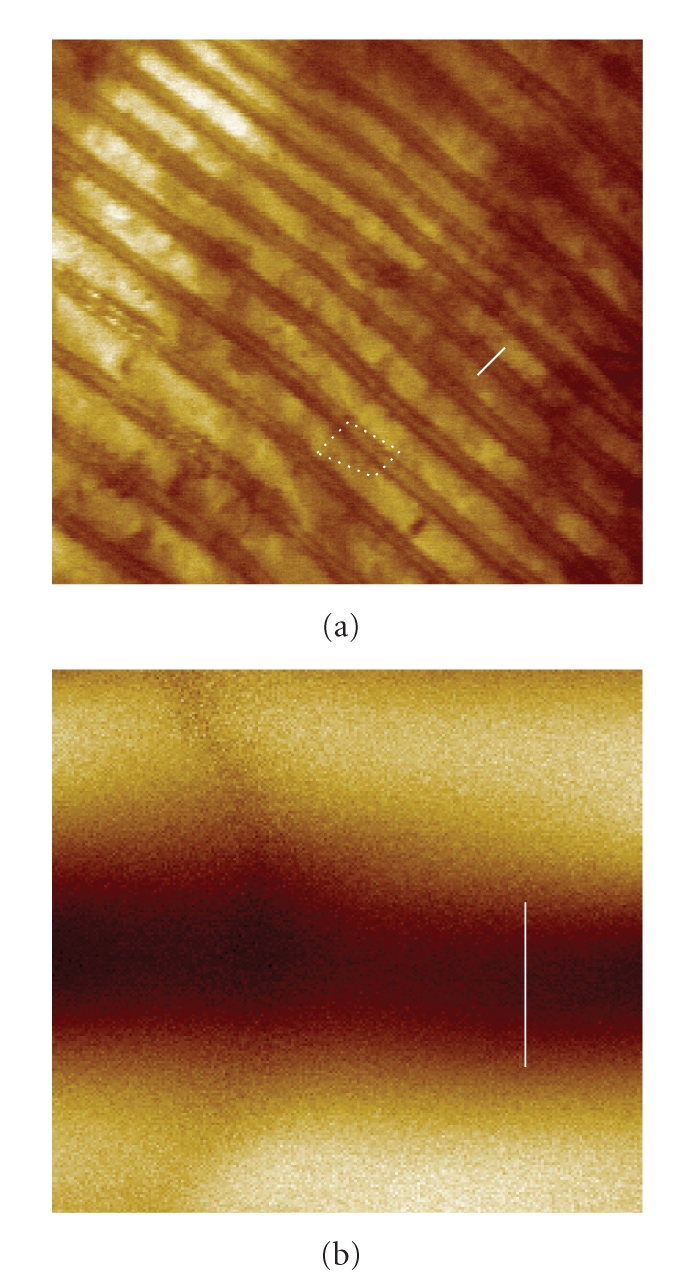
AFM topographic scans showing surface structure of the cuticle; (a) is a map from the scan on a larger area of about 3 × 10^5^ square pixels (b) is a scan on a single pixel of about 2.5 × 10^6^ nm^2^. Doted lines on (a) represents the region shown by (b). scale bars ∼ 0.5 nm.

Figures 3(a) and 3(b) are AFM scans showing surface structural characteristics of the cuticle sample. The AFM topographic scan shows that the cuticle has a highly oriented surface topography. The interstitial regions between the ridges represented by dark area are cavities on the membrane having an approximate width of 0.5 nm. This results are similar to AFM studies on the surface of cellulose [30] which showed that surface structural characteristics of native cellulose consists of planes of cellulose molecules which are regularly interspaced at about 0.53 nm.

**Figure 4:**
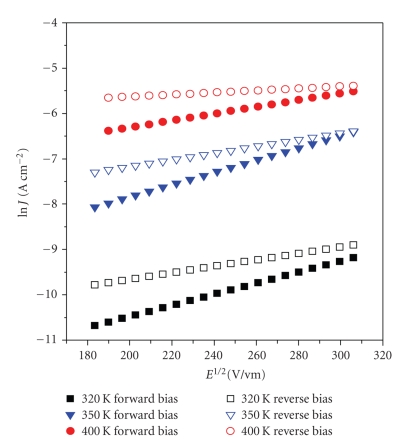
Semilogarithmic plots of ln* J* versus *E* ^1/2^ for the high field of 10^4^–10^5^ V/cm in forward bias (increasing electric field) and reverse bias (decreasing electric field) and poling temperature range 320 K–370 K (R-reverse bias; F-forward bias).

Forward and reverse bias current density-electric field dependence at high fields (10^4^–10^5^ V/cm) are displayed in Figure 4. The current levels in the reverse bias are higher than forward bias, showing relatively linear relations of ln *J versus E^1/2^*. This behavior may be interpreted either in terms of Schottky effect which is a field lowering of interfacial barrier at the blocking electrode or by Poole-Frenkel effect which is due to thermal excitation of trapped charges via field assisted lowering of trap depth [19]. Expressions for these processes are shown in (1) and (2) [20]:

(1) $$J_{S}=J_{\text{SO}}\exp\;\left[\left(\beta_{S}E^{1/2}\right)/kT\right]$$

for the Schottky effect and

(2) $$J_{\text{PF}}=J_{\text{PFO}}\exp\;\left[\left(\beta_{\text{PF}}E^{1/2}\right)/kT\right]$$

for the Poole-Frenkel effect. 

*J*_SO_  and *J*_PFO_ are preexponential factors, *β*_*S*_ is the Schottky coefficient,  *β*_PF_ is the Poole-Frenkel coefficient, and *E* is the electric field. Theoretical values of Schottky and Poole-Frenkel coefficient are related by (3):

(3) $$\beta_{S}=\frac{e^{3}}{4\pi \varepsilon \varepsilon_{0}}=\frac{\beta_{\text{PF}}}{2}.$$

With the assumption that the value of *ε* for our samples is 3.0, the reported value for ethyl cellulose [31], the theoretical values of *β*_*S*_ and *β*_PF_ were obtained from (3) and found to be 3.51 × 10^−24^ J V^1/2^ m^1/2^ and 7.01 × 10^−24^ J V^1/2^ m^1/2^, respectively. Experimental values of **β** obtained from slopes (linear regression parameters) of linear fit plots of ln *J* *versus E^1/2^* (Figure 4) at different temperatures are listed in Table 2. The large discrepancy in experimental values of **β** listed in Table 2 and theoretical values of *β*_*S*_ and *β*_PF_ leads to a conclusion that current transport mechanism in our samples governing the high field at a temperature range of 320–370 K cannot be explained in terms of Shottky or Poole-Frenkel emission.

**Figure 5:**
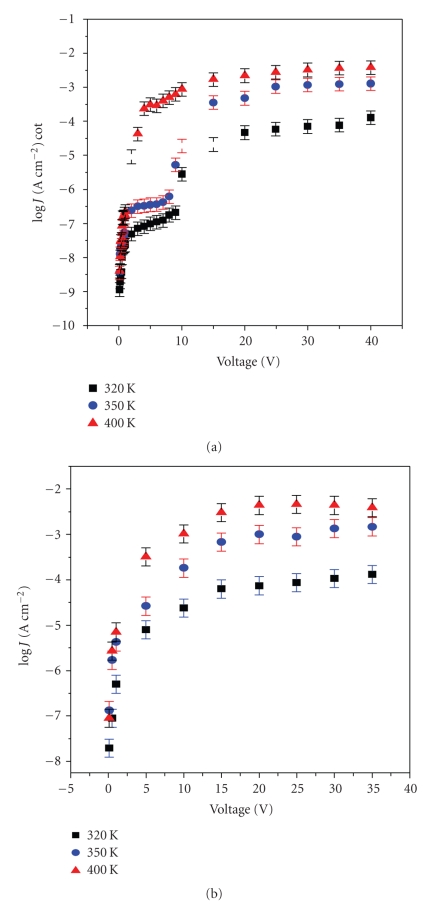
(a) Semilogarithmic plots of log *J versus V* as a function of poling temperature in the forward bias regime. (b) Semilogarithmic plots of log* J* *versus V* as a function of poling temperature in the reverse bias regime.

Figures 5(a) and 5(b) show *J-V* characteristics over a temperature range of 320–350 K for the forward bias and reverse bias, respectively. Figure 5(a) shows that forward bias characteristics has two regions: low voltage region below a threshold voltage (*V*_th_) where current density follows ohms law relations (ohmic region), and higher voltages region, above *V*_th_ at which, there is a pronounced power-law behavior given by *J α V* ^*n*^ where *n* ≈ 1.8 ± 0.3 (SCLC region). *V*_th_ has negative temperature coefficient as noticed in Figure 5(a) where it decreases as temperature increase—a case that is identical to tunneling of charge carriers through the entire metal-insulator-metal junction for a structure with multiple tunnel barriers in the Coulomb blockade regime [32]. At a poling temperature of 400 K, *V*_th_ decreased leading to a smooth transition from low ohmic region to SCLC. Existence of *V*_th_ elucidates electrical switching of the material from high impedance region where the current densities are very low, to low impedance region which allows large currents to flow. Figure 5(b) shows that our samples remained in the low impedance region even as the voltage was decreased (reverse bias) leading to high levels of leakage current in the reverse bias than in the forward bias (see Figure 4). This can also be explained in terms of built up of image charges at high field of the reverse bias which lowers potential barrier at the metal-polymer junction hence allowing higher current to flow in the reverse bias [21]. This phenomenon of electrical switching with memory has also been observed in some synthetic polymers [33–36] and has been used as active cells in modern nonvolatile memory chips [37]. Polymers which exhibit electrical switching and memory effect can also be applied in electrical switching circuit and in gas sensing [36]. Electrical switching is explained in terms of formation of semiquinones and quinoid radicals [3] and in terms of SCLC. As shown in Figure 5(a), the low current densities at low voltages is due to charge capture in traps (intrinsic charges) present in the cuticle. Increase in bias voltage results in an increase in injected charge, thereby filling the limited traps. This injection is provided by tunneling of the electrons (holes) into the conduction (valence) bands of the polymer. Injection of charges from metal electrode causes a reduction in the number of traps which, leads to a rapid increase in effective carrier mobility and therefore a rapid power-law increase in current. At sufficiently high injection levels, all the traps are filled reaching the trap filled limit (TFL) and consequently the current becomes SCLC. The total trap concentration *N_t_* in the cuticle sample is defined in (4) [31]:

(4) $$N_{t}=\frac{2\varepsilon \varepsilon_{0}}{ed^{2}}V_{\text{TFL}},$$

where *e* is the electronic charge *ε* relative permittivity of the material *ε*_0_ is the permittivity of free space, *d* is thickness of sample (4 *μ*m) and, *V*_TFL_ is the upper limit of the voltage at which sufficient charge has been injected into the insulator to fill the traps. Equilibrium carrier concentration *n*_0_ can be obtained from the expression (5) [38]:

(5) $$n_{0}=\frac{9}{8}\frac{\varepsilon \varepsilon_{0}}{ed^{2}}V_{\text{tr}},$$

where *V*_tr_ is the voltage at which the transition from ohmic to SCLC behavior takes place. Using experimental values of *V*_TFL_ = 15 V and *V*_tr_ = 6 V obtained from *J-V* curve at 320 K (Figure 5(a)), the values of *N_t_* and *n*_0_ were calculated from (4) and (5). Current density at low voltage (the ohmic conduction region) is given by (6):

(6) $$J=\frac{p_{0}e\mu V}{d},$$

where *p*_0_ is the concentration of thermally activated holes expressed as shown in (7):

(7) $$p_{0}=N_{V}\exp\;\left(-\frac{E_{d}}{kT}\right).$$

**Figure 6:**
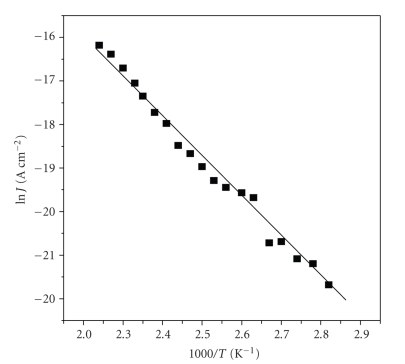
Semilogarithmic plot of ln* J versus 1/T* at temperature range of 350–440 K and applied voltage of 0.9 V.

Current density then becomes

(8) $$J=\frac{N_{v}e\mu V}{d\exp\;\left(E_{d}/kT\right)},$$

where *N_v_* is effective density of states, *e* is electron charge, *μ* is charge mobility, *V* is the applied voltage, *d* is the sample thickness, *E_d_* is the ionization energy of donor charges, *k* is the Boltzmann's constant, and *T* is the temperature. By plotting ln   *J* versus 1000/*T* it is possible to determine *μ* and *E_d_*. Variation of ln *J* with 1000/*T*, at a voltage of 0.9 V (ohmic region) is shown in Figure 6. Assuming that *N*_*v*_ = 10^19^ cm^−3^ which is within the range of the effective density of states for thin films of other organic semiconductors [39], the value of *p *_o_, *μ*, and *E_d_* are determined from (7) and (8). These electrical parameters, *p *_o_, *μ*, and *E_d _* being obtained from analysis of temperature dependence of ohmic region and *N_t_*, *n*_0_ obtained from analysis of SCLC regime are listed in Table 3. The low charge mobility and the electrical parameters shown in Table 3 are comparable to same parameters calculated for other organic semiconductors [31, 38].

**Figure 7:**
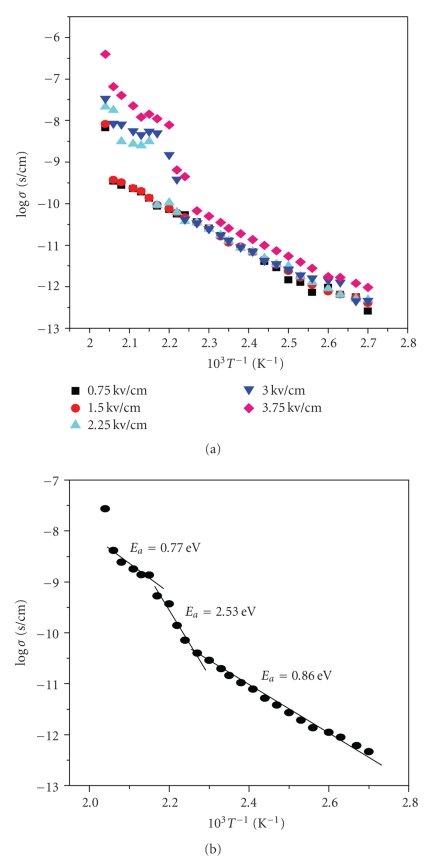
(a) Arrhenius plots showing Variation of **σ* versus 1/T* at different electric fields. (b) Average Arrhenius plot showing activation energy at different temperature regions at an applied electric field of 2.25 Kv/cm (0.9 V).

Thermal activation plots (log *σ versus* 10^3^ */T*) for different polarizing fields were plotted to analyze the effect of temperature on conductivity of the samples (Figure 7(a)). The observed dependence can be described by the Arrhenius equation (9):

(9) $$\sigma =\sigma_{0}\exp\;\left(\frac{-E_{a}}{kT}\right),$$

where **σ** is conductivity, **σ*_o_* the preexponential factor, and *E_a_* the activation energy. Conductivity was obtained from the relations in (10):

(10) $$\sigma =\frac{I}{V}\frac{L}{A},$$

where *I* is measured current, *V* is measured Voltage, *L* is the thickness of the samples (≈4.0 × 10^−4^ cm), and *A* is the electrode active area. Activation energy, *E_a_* was calculated from the average curve (Figure 7(b)) of log *σ versus* 10^3^ */T* at different temperature regions.

**Figure 8:**
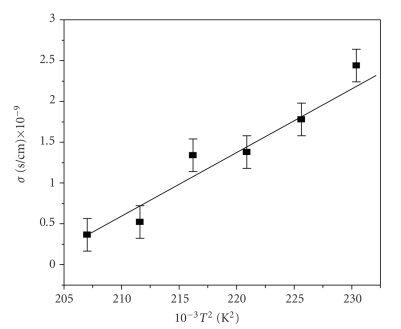
Plot of **σ** versus *T* ^2^ in the range 460–480 K where *E*_*a*_ ∼ 0.77 eV.

Figure 7(b) shows three regions of conduction indicated by different activation energies. The increase in the electrical conductivity and the decrease in the activation energy for the films sample could be attributed to the amorphous-crystalline transformation [40]. A polycrystalline film material contains a large number of microcrystallites with grain boundaries between them. At the grain boundary of each of the crystallites incomplete atomic bonding can act as trap centers. These trap centers trap charge carriers at the grain boundaries, and hence a space charge can be built up locally [40]. Increase of temperature, increases probability of thermal ionization of the trapping centers thus causing a shift in the quasi-Fermi level and the possibility of new trap states in the mobility gap [31, 40]. Electrical conduction mechanism at the temperature region where activation energy is low, (*E*_*a*_ ∼ 0.77 eV) and where conductivity is almost constant (Figure 7(b)) is by tunneling of charge carriers across the grain boundaries. This is supported by almost linear variation of **σ** versus *T* ^2^ as shown in Figure 8. 

The log **σ* versus* 10^3^ */T* variation at 350 < *T* < 440 K region is linear (Figure 7(b)). Investigation of conduction mechanism in this temperature range was made on the basis that tunneling or hopping mechanisms can predominate. Temperature-dependence data in Figure 7(b) were reused to plot **σ** versus *T* ^2^, and ln (*σT* ^1/2^) versus *T* ^−1/4^. From this analysis, we noted that tunneling conduction mechanism in the range of 350–440 K was inadequate due to nonlinear variation of **σ** versus *T* ^2^. Thermionic emission was also ruled out because this mechanism does not take place when the applied voltage (*V*) is greater than 0.8 V [21], and at low activation energy [41]. 

**Figure 9:**
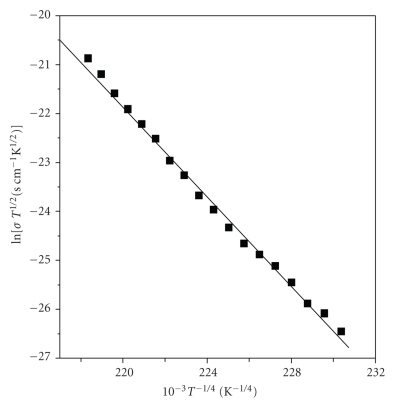
Plot of ln (*σT* ^1/2^) versus (*T* ^−1/4^) for NFSC within a temperature of 350 K–440 K and applied electric field of 2.25 Kv/cm.

Figure 9 shows linear variation of (*σT* ^1/2^) versus *T* ^−1/4^ thus indicating that VRH conduction process can predominate in this temperature range 350–450 K [41]. A good fit of conductivity-temperature data to the expression in (11):

(11) $$\sigma T^{1/2}=\sigma_{0}\exp\;\left[-{\left(\frac{T_{0}}{T}\right)}^{1/4}\right]$$

is necessary for applicability of VRH model. The preexponential factor **σ**_0_ and the degree of disorder (Mott temperature) *T*_0_ are related to the density of states *N(E_F_),* and the inverse-fall-off length of the wave function of a localized state near the Fermi-Level **α** by the following relations [12]:

(12) $$\sigma_{0}=\frac{3e^{3}\upsilon_{ph}}{{\left(8\pi k\right)}^{1/2}}{\left[\frac{N(E_{F})}{\alpha }\right]}^{1/2},$$

(13) $$T_{0}=18.11\frac{\alpha^{3}}{kN\left(E_{F}\right)}.$$

To check the validity of the VRH model, ln (*σT* ^1/2^) versus *T* ^−1/4^ variation was plotted (Figure 9). *T*_0_, and *N(E_F_)* were determined from (13) using the slope in Figure 9, and assuming that *α* ≈ 3 Å^−1^ which is approximately equal to inverse length of unit cell (monomer) of native cellulose [42–44]. Other Mott parameters, the distance *R* and average energy *W* were determined at *T* = 400 K by using the following relations in (14) and (15) [23]:

(14) $$\begin{array}{cc} R & ={\left[\frac{9}{8\pi \alpha kTN(E_{F})}\right]}^{1/4}, \end{array}$$

(15) $$\begin{array}{cc} W & =\frac{3}{4\pi R^{3}N\left(E_{F}\right)}. \end{array}$$

The obtained Mott parameters are listed in Table 4 which shows that the product *αR* and the average energy, *W,* satisfy Mott's requirements *(αR* > 1, *W* > *kT*) for variable range hopping at this temperature range. Degree of localization of the carriers in the trap states indicated by *αR* > 1, shows that the charges are highly localized. Table 5 shows the variations of the Mott parameters with temperature in our samples. It is evident from this table that *αR* > 1 and *W* > *kT*, which agrees with Mott's condition for variable range. It can also be noted from Table 5 that when the temperature decreases, the average energy *W* decreases and the average distance *R* increases, supporting the fact that when the phonon energy is insufficient (low temperature), carriers will tend to hop larger distances in order to locate in sites which are energetically closer than their nearest neighbours. 

## 4. Conclusion

DC conductivity measurements were performed at a temperature range of 320 K–450 K. Conductivity increased with temperature. Activation energy within a wider range of temperature was about 0.86 eV. Charge mobility analyzed from *J-V* curves was found to be 4.06 × 10^−5^ (m^2^V^−1^s^−1^). Analysis of current density-electric field dependence showed that both Schottky and Poole-Frenkel emission cannot explain conduction mechanism in the high fields where SCLC was seen to be present. Plots of DC conductivity versus temperature were used to investigate Mott's VRH conduction model at a temperature range of 320–440 K. This model was originally developed for amorphous silicon by Mott and Davis. When applied to conducting polymers, it assumes that electron transport originates from localized or fixed states within the polymer chain. The charge transfer between the chains takes place by phononassisted hopping between two localized states. Analysis of semilogarithmic plots of ln*(JT* ^1/2^) *versus T* ^−1/4 ^ gave the following Mott parameters: the degree of disorder *T_o_* was in the order of 10^10^, the density of states *N*(*E*_*F*_) ≈ 10^19^  (eV^−1^cm^−3^) while the distance *R* ≈ 10^−7^ cm and energy (*W*) were in the range of 0.72 eV–0.98 eV. The product *αR* and the average energy, *W*, satisfy Mott's requirements *(αR* > 1, *W* > *kT*) for variable range hopping in this temperature range. The study has shown that the cuticle is a promising alternative to synthetic polymers used in electrical switching applications. Due to environmental concerns posed by nonbiodegradable synthetic polymers, exploring this material would provide information on how to use it in addressing environmental problems and as a precursor in the design of novel materials with electrical functionality such as the modern nonvolatile memory chips, biosensors, and biological transistors.

## Figures and Tables

**Table 1:** FT-IR frenquencies (cm^−1^) of cellulose, Nandi flame seed cuticle (NFSC), and pectin (PGA-polygalacturonic acid) *Wilson et al. [29].

Cellulose	NFSC	Pectin*	Band assignment*	Comments*
		1745	}*ν*(C=O)	
	1725			PGA, ester
1712				
1647			}*δ*(HOH)	Adsorbed water
1644		1640		
	1602	1605	*ν*_as_(COO^−^)	PGA, carboxylate
		1444	*δ*(CH)*δ*(CH_2_)	PGA, ester
1424	1422		}*ν*_s_ (COO^−^)	PGA, Carboxylate
		1419		
1365	1368	1368	}*δ*(CH_2_), *ν*(CC)	
1351	1352			
1334	1332	1335	*δ*(CH), ring	
1318	1316		}*δ*(O–C)	
1277	1278			
1230	1232			
1156	1152	1150	*ν*(C–O–C), ring	Glycosidic link
1109	1106	1107	*ν*(CO), *ν*(CC), ring	
1050	1052	1055	*ν*(CO), *ν*(CC), *δ*(OCH)	
1032		1033	*ν*(CO), *ν*(CC), *ν*(CCO)	
	1022	1018	*ν*(CO), *ν*(CC), ), *δ*(OCH), ring	PGA, Pectinate
	1006	1008	*ν*(CO), *ν*(CC), ), *δ*(OCH), ring	PGA, Pectate
	981	972	OCH_3_	
	954	963	CO, *δ*(C=O)	

**Table 2:** Values of **β** obtained from experimental data.

Temperature (K)	Experimental *β* (Jm^1/2^ V^1/2^) values	
	Forward bias	Reverse bias
320	5.65 × 10^−23^	3.17 × 10^−23^
350	6.31 × 10^−23^	3.56 × 10^−23^
370	3.44 × 10^−23^	0.93 × 10^−23^

**Table 3:** Electrical parameters analyzed from *J-V* characteristics at 320 K.

Parameter	Value
*E_d_* (eV)	0.827
*μ* (m^2^ V^−1^ s^−1^)	4.06 × 10^−5^
*P_o_* (m^−3^)	9.35 × 10^10^
*n_o_* (m^−3^)	6.22 × 10^19^
*N_t_* (m^−3^)	4.66 × 10^21^

**Table 4:** Mott parameters at temperature range of (320–440 K).

Mott parameters	Value
*T*_0_ (K)	4.58 × 10^10^
*N_(EF)_* (eV^−1^cm^−3^)	9.04 × 10^19^
**α** (cm^−1^)	3.0 × 10^8^
*R* (cm)	1.44 × 10^−7^
*W* (eV)	0.89

**Table 5:** Variation of Mott parameters at temperature range of 300–450 K.

*T* (K)	*R* (cm^−1^)	*W* (eV)	*kT* (eV)	*αR*
300	1.54 × 10^−7^	0.72	0.026	41.6
350	1.49 × 10^−7^	0.81	0.030	40.1
400	1.44 × 10^−7^	0.89	0.034	38.7
450	1.39 × 10^−7^	0.98	0.039	37.6
